# Automatic detect lung node with deep learning in segmentation and imbalance data labeling

**DOI:** 10.1038/s41598-021-90599-4

**Published:** 2021-05-27

**Authors:** Ting-Wei Chiu, Yu-Lin Tsai, Shun-Feng Su

**Affiliations:** Department of Electrical Engineering in National Taiwan University of Science and Technology, Taipei, 106 Taiwan

**Keywords:** Lung cancer, Cancer screening, Computer science, Image processing, Machine learning

## Abstract

In this study, a novel method with the U-Net-based network architecture, 2D U-Net, is employed to segment the position of lung nodules, which are an early symptom of lung cancer and have a high probability of becoming a carcinoma, especially when a lung nodule is bigger than 15 $\mathrm{mm}^2$. A serious problem of considering deep learning for all medical images is imbalanced labeling between foreground and background. The lung nodule is the foreground which accounts for a lower percentage in a whole image. The evaluation function adopted in this study is dice coefficient loss, which is usually used in image segmentation tasks. The proposed pre-processing method in this study is to use complementary labeling as the input in U-Net. With this method, the labeling is swapped. The no-nodule position is labeled. And the position of the nodule becomes non-labeled. The result shows that the proposal in this study is efficient in a small quantity of data. This method, complementary labeling could be used in a small data quantity scenario. With the use of ROI segmentation model in the data pre-processing, the results of lung nodule detection can be improved a lot as shown in the experiments.

## Introduction

**Figure 1:**
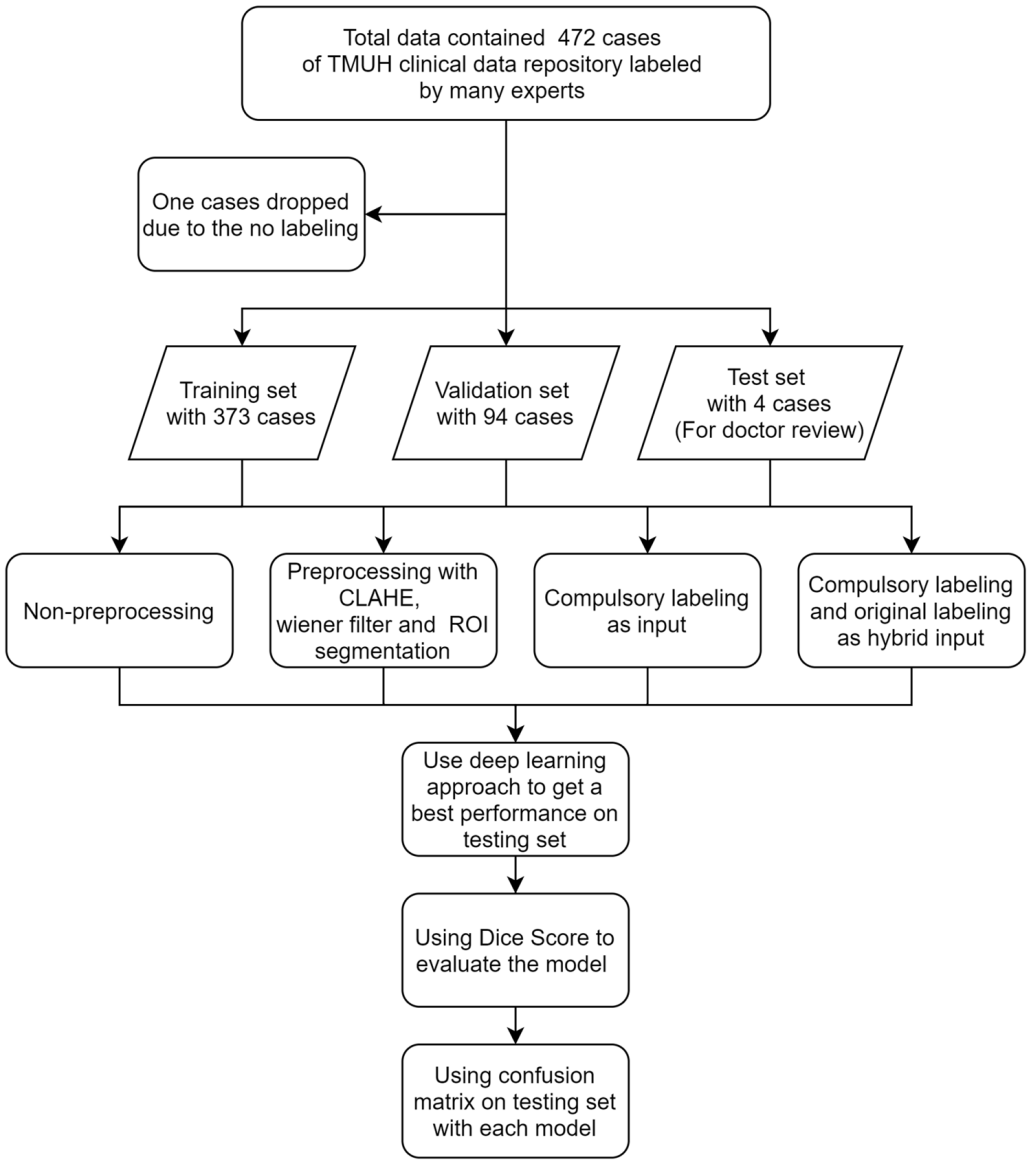
Flowchart of the processing in data split and model evaluation.

Lung cancer is ranked the first place at leading ten causes of death in Taiwan for years. World Health Organization (WHO) has appealed that early detection of cancer could greatly increase the chances of successful treatment. One effective way of diagnosis is CT (computed tomography) scan. CT scan is composed of many X-ray images taken from different angles and is an effective medical imaging procedure. CT has a high diagnosis value for lung tissues. It has nice results without a radio contrast agent. CT images are known to be able to provide better details than X-rays do on organs, bones, soft tissue, and blood vessels. However, Lung nodule, which is a soft tissue in the lung and is an early symptom of lung cancer, but is difficult to be detected by doctors. Due to the difficulty, misdiagnosis commonly happens in the diagnosis of this disease. In this study, deep learning-based recognition is considered to help doctors to make a diagnosis precisely. In doctors’ insight, to diagnose possible nodules is time-consuming and hard to do. The precision of the medical diagnosis is an important issue in the medical field. It will cause a serious result once a diagnostic bias happens in a case. This study is viewed as an auxiliary diagnosis through artificial intelligence techniques. However, there are lots of related approaches about lung nodule detection. Like those in papers^1–3^. The main differences between them and our approach are the method used and the data structure considered. There are 472 CT image cases of patients in our database. The data process is shown in Fig. 1. The pre-processing techniques used are similar to those of the other approaches, but the labeling used is inverted. Then, the original label and the invested label are concatenated together as the input to the neural network used. Convolutional Neural Network (CNN) is used to recognize lung nodules in CT images. U-net^4^ is a usually used segmentation model in medical image segmentation. U-net shows good results in many applications^5–7^ recently. Due to the connectivity, 3D U-net^8^ stacking many 2D medical images together is also used in medical image segmentation in recent years. Because of the setting of memory problem scenario, 2D U-net is considered as our model in the study. Res U-net^9^ is combined with a basically U-net and Residual blocks^10^. It will have more connectivity in different convolutional layers. In this study, Res U-net is used to conduct the CT image segmentation to get the position of pulmonary first, and then U-net is used to get the position of lung nodules. A serious problem of considering deep learning for all medical images is imbalanced labeling between foreground and background. The same problem occurs in this study. The lung nodule is the foreground and accounts for a lower percentage in a whole image. The results show that imbalanced data de-biasing is a difficult task in the medical field. It is worthwhile to mention that heterogeneity is a serious problem and needs to be solved in deep learning. A method of labeling data in two different ways is proposed to get over heterogeneity. Complementary labeling the foreground is an efficient way of reducing heterogeneity. The evaluation chosen in this study is dice coefficient loss which is a typical loss function in the image segmentation field. In the testing, results show that complementary labeling is a method to have a better result than that of using original labeling in small data quantity. Since it is hard to label the medicine images, the quantity of medical images labeling is usually not sufficient. It well-known that to resolve the problem of small data quantity is hard. Semi-supervised learning^11^ for auto-labeling is one efficient way of resolve the lack of labeling problem. In addition, using complementary labeling is also another efficient way. In this study, a net is used to perform the ROI segmentation with CT images to get the position of pulmonary. It can be viewed as the attention model for the process. With the use of ROI segmentation model in the data pre-processing, the results of lung nodule detecting can be improved a lot as shown in the experiments.

## Methods

### Data description

We confirm that all methods were carried out in accordance with relevant guidelines and regulations. This study was approved by Taipei Medical University Hospital Joint Institutional Review Board and is not a retrospective study. All of the patients signed the informed consent of identifying information and image publishing. Furthermore, all of the records from patients with pathologically was confirmed and retrospectively reviewed by the final pathological confirmation or clinical diagnosis from 2016 to 2019. CT reports were searched for target patients by initially using the keyword “CT” and “nodule”. After achieving the first round filtered cases, cases with keyword “nodule”, “opacity”, “GGO (ground-glass opacity)”, “adenocarcinoma”, “granuloma”, “metastasis“ and “cancer” in type section and “pleural”, “hilum”, “pulmonary”, “lung”, “RUL”, “RLL”, “EML”, “LLL”, “LUL” in position section would be kept by manually CT report screening. It is noted that the CT reports with “shadow”, “emphysema”, “pneumonia”, “pneumonitis”, “cysts”, “fibrotic foci”, “inflammation”, and “consolidative patchy” were not included. The inclusion criteria for the study were as follow: (1) patients were scanned with routine CT using a slice thickness of 5 mm; (2) diagnosis without distant metastasis was confirmed by surgery and pathology; (3) Only the last CT scan before surgery or biopsy was chosen. (4) The diameter of each nodule was smaller than 30 mm. Under above criteria, a total of 457 cases (220 women, 236 men; average age, $65\pm 13$) with 472 lung nodules were enrolled in the study.

The all chest CT images were taken under free-breathing condition with supine position on the scanning bed for all the patients. CT scanners that produced by these manufacturers (GE Medical Systems, Philips Medical Systems, Siemens) were used for the acquisition of those CT images with 110–120 kV and 10–20 mA. The image slice matrix was 512 $\times$ 512, with slice thickness of 5 mm and the pixel spacing of 0.168 $\times$ 0.168 $\text {mm}^2$.

There are two nodule morphologies, segmentation and semantic features in this dataset. All were discussed by three radiological doctors with 10 to 20-year radiological experiences with consensus. These CT images are analyzed mainly on the lung window (the range of HU values from −1400 to 400). There are two steps in nodule segmentation. The first is to use a semi-automatically segmentation way to get the target nodules on commercial software (IntelliSpace Discovery, Netherlands, Philips Healthcare) and then to store into a DICOM format. The second one are the contours which were segmented and would be checked again by another doctor. The nodule contour would be modified by freehand drawn using a self-developed software running on Matlab (r2018b, The MathWorks, USA) if necessary. The data format is DICOM with de-identification. DICOM (Digital Imaging and Communication in Medicine) is a general communication protocol. It can integrate the medical applications from multiple manufacturers such as scanners, servers, workstations, printers, etc. DICOM is widely used in hospital and be used in local clinics and dentist clinics.

### Pre-processing

In our implementation, the data format is transferred from DICOM to NIfTI. NIfTI (Neuroimaging Informatics Technology Initiative) is an open file format. It was developed for neuroimaging at the first but now is widely used in brain images and other medical images. The feature of this format has two affine coordinates linked to its value pixel (i,j,k) and its position space (x,y,z).

**Figure 2:**
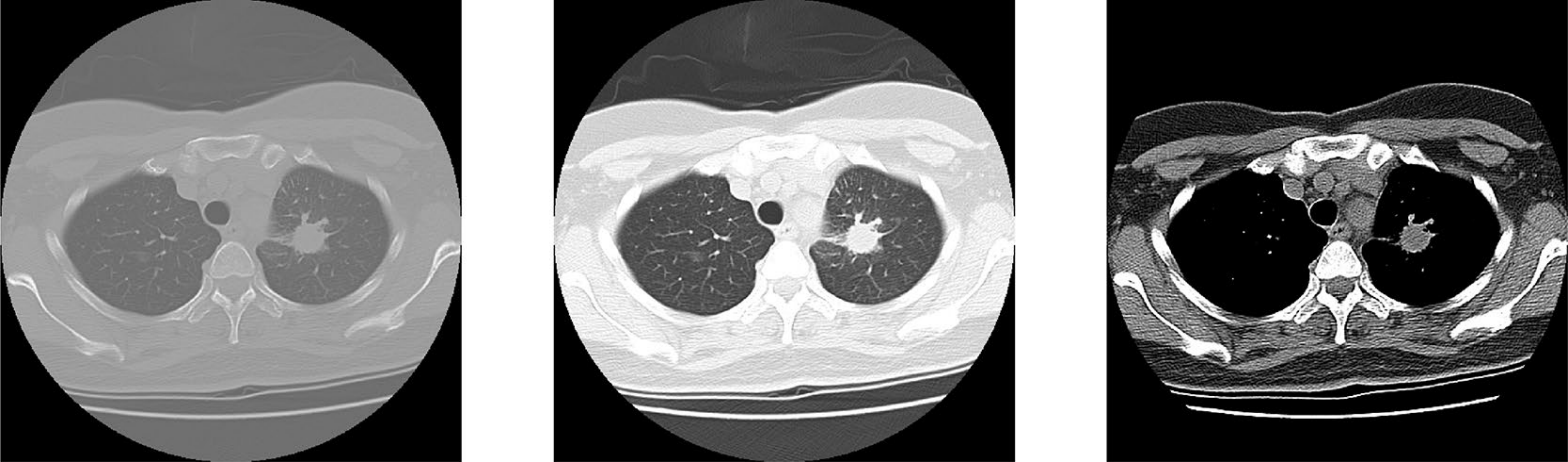
From left to right: Original, lungwindow and softtissue.

Each scan of the data is a 3D medical image of a resolution in 512 $\times$ 512 that spanning multiple slices about 100 slices. NIfTI format is used because NIfTI is for 3D images processing. The DICOM format will consume more memories because of the data space. CLAHE^12^ (Contrast Limited Adaptive Histogram Equalization) is used to balance the contrast in CT images. The different hounsfield (HU) in CT images are divided into three different windows, original, softtissue, and lung window in this study as shown in Fig. 2. The mainly difference between them is the contrast and image detail. The hounsfield center value and width of lung window and softtissue is −600, 1500 and 50, 400 respectively. The lung window is selected as our input images because this is the most common hounsfield range used in clinic diagnosis of lung images. The hounsfield value parts of the body is shown in Table 1. After the sampling, images are resized from 512 $\times$ 512 to 256 $\times$ 256 to decrease the memory consumption. Doing normalization is the next step to decrease the computing cost. Then CLAHE is used to adjust the contrast to make the nodule more obvious.

**Table 1:** HU value in parts of the body.

Substance	HU
Air	−1000
Fat	−120 to 90
Soft tissue on contrast CT	+100 to +300
Lung	−700 to −600
Bone	+300 to +400
	+1800 to +1900
Water	0

### CLAHE

**Figure 3:**
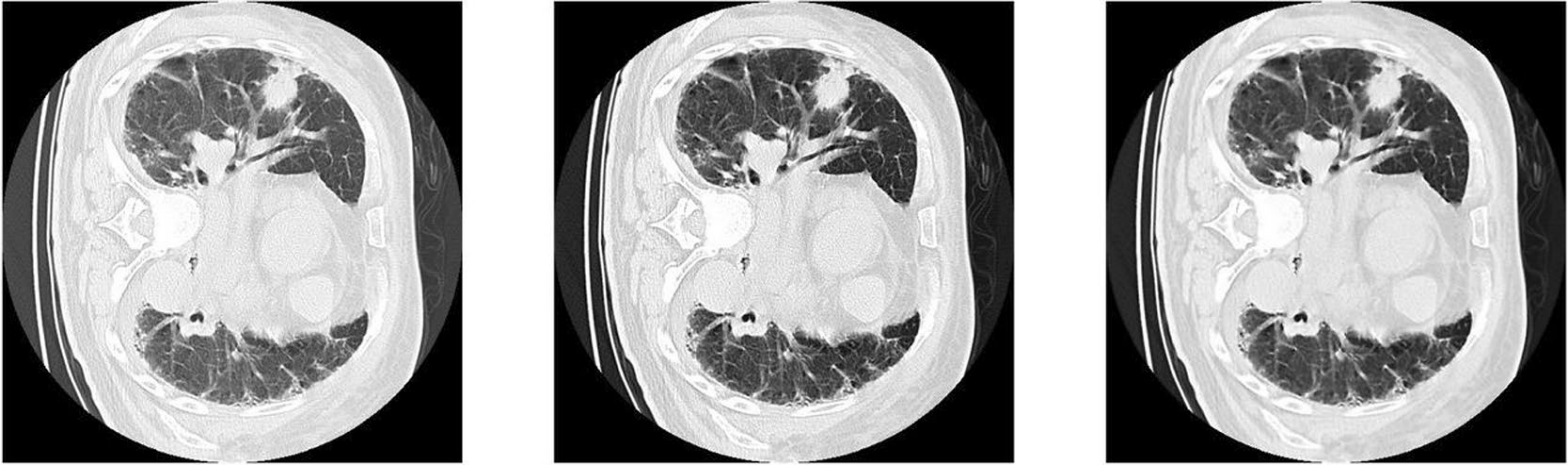
From left to right, Original CT image, With CLAHE, With CLAHE and Wiener.

CLAHE (Contrast Limited Adaptive Histogram Equalization) is widely used in image processing. It differs from AHE^13^ (Adaptive Histogram Equalization). The traditional adaptive histogram equalization has a tendency to over amplify noise in relatively homogeneous regions in images. It is because the histogram in these regions is highly concentrated. As a result, AHE may cause noise to be amplified in near-constant regions. CLAHE can limit the concentration of the histogram. The advantage of CLAHE is that it will not discard the part of the concentrated histogram. Instead, it keeps them and redistributes the exceed histogram equally among all histogram bins. As mentioned in the study of^14^, using the Wiener filter can efficiently decrease the noise in images. The PET/CT images are prone to constant power additive noise. Figure 3 shows the performance of pre-processing with the wiener filter and CLAHE in TMUH dataset.

### U-net

**Figure 4:**
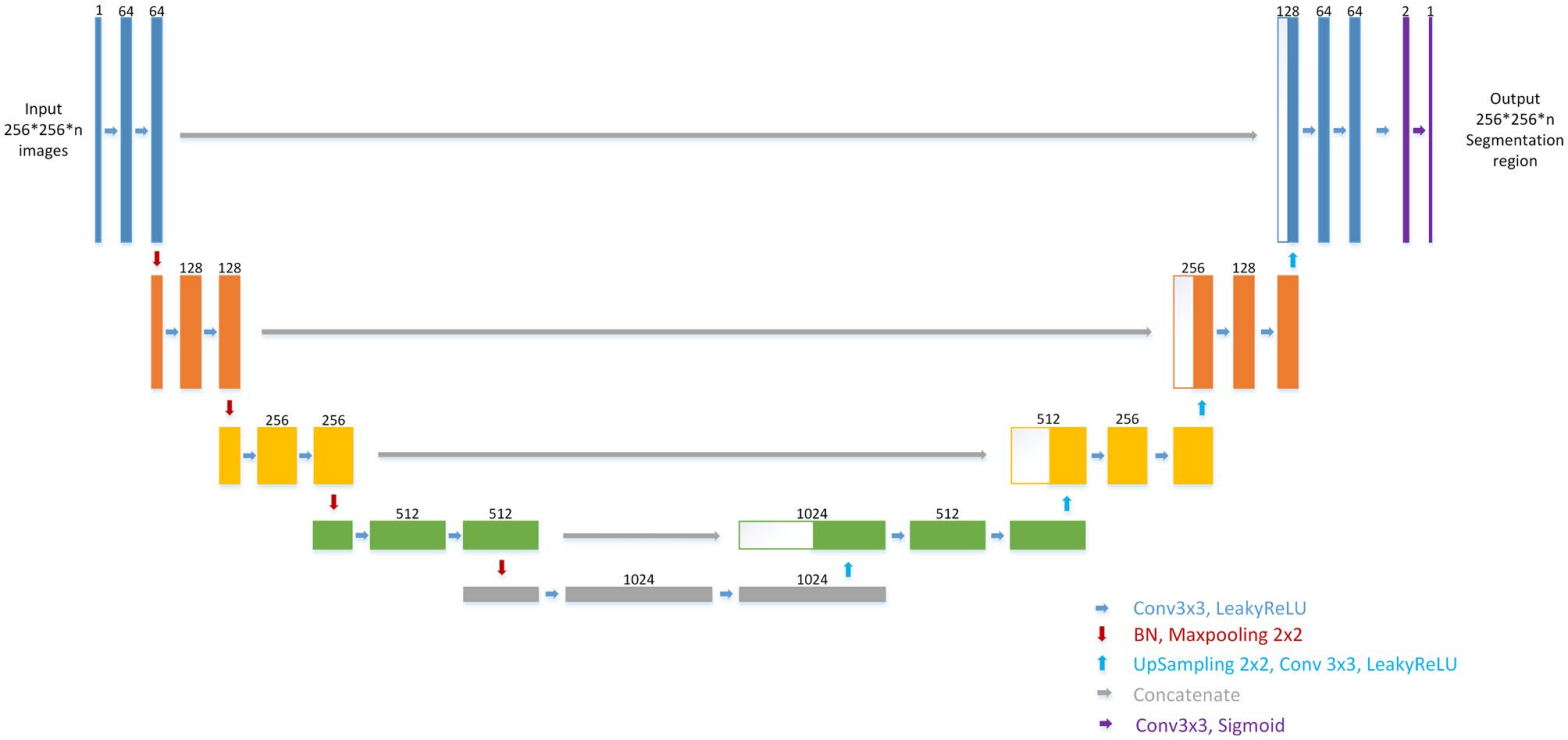
U-net architecture. Each box corresponds to a multi-channel feature map. The number of channel shown on top of the box. The bottom right corner shows the meaning of the arrows. The different between ours and original U-net is we use LeakyRelu(alpha = 0.3) as activation function in each convolution layer.

U-net is an encoder-decoder deep learning model which is known to be used in medical images. It is first used in biomedical image segmentation. U-net contained three main blocks, downsampling, upsampling, and concatenation. As shown in Fig. 4, U-net is well known for its architecture. Some literature called the architecture as the encoder- decoder architecture. Since the shape of the net looks like the uppercase English letter U, it is named as U-net. As shown in the figure, each box corresponds to a multi-channel feature map. The number of channels shown on top of the box. The bottom right corner shows the meaning of the arrows. However, He Initialization is adopted as initial weight without pre-training in this model and the difference between the net used in our study and the original U-net is that the LeakyRelu with alpha = 0.3 is used as an activation function in each convolution layer. The important difference between U-net and other segmentation net is U-net used a totally different feature fusion way: concatenation. It concatenates the feature channel together to get a feature group. It could decrease the loss of features during convolution layers. And the training result would be better than without it.

### ROI segmentation model

**Figure 5:**
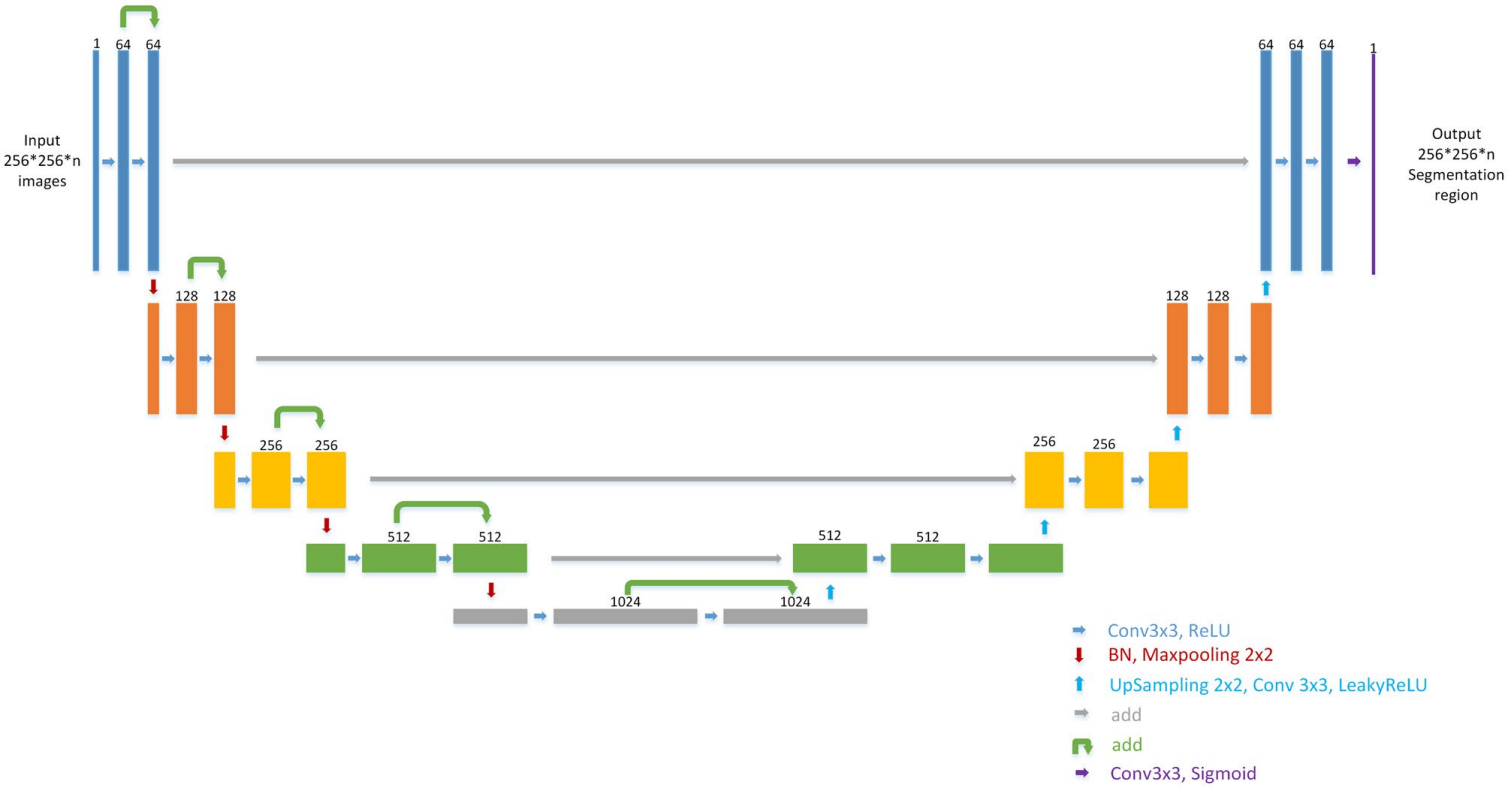
Res U-net architecture. This net is used to do the ROI segmentation with CT image to get the position of pulmonary. Each box corresponds to a multi-channel feature map. The number of channel shown on top of the box. The bottom right corner shows the meaning of the arrows.

Lots of ways are considered to get the position of lung nodules. Initially, no pre-processing is considered on the images. The result seems not good. In the study^15–17^, they used thresholds to get the position of the object to be recognized. In the paper^18^, they used bounding boxes to get the positions of nodules. In our study, the deep learning-based ROI segmentation model used is as shown in Fig. 5 and is to get the position of the whole lung. This net is used to perform the ROI segmentation with CT images to get the position of pulmonary. It can be viewed as the attention model for the process. Each box in Fig. 5 corresponds to a multi-channel feature map. The number of channels shown on top of the box. The bottom right corner shows the meaning of the arrows. The segmentation labels are gotten from the open data LCTSC (Lung CT Segmentation Challenge) by using ROI Segmentation Res-Unet of which the architecture is somewhat like Res U-net to accomplish ROI segmentation. Res U-net is Unet with residual blocks which is first proposed in 2015 by Kaiming He and those residual blocks can efficiently decrease training loss as the training epoch goes on. The segmentation labeling data are augmented by each case to 300. With the use of ROI segmentation model, the results of lung nodule detecting can be improved a lot as shown in the later experiments.

### Complementary labeling

**Figure 6:**
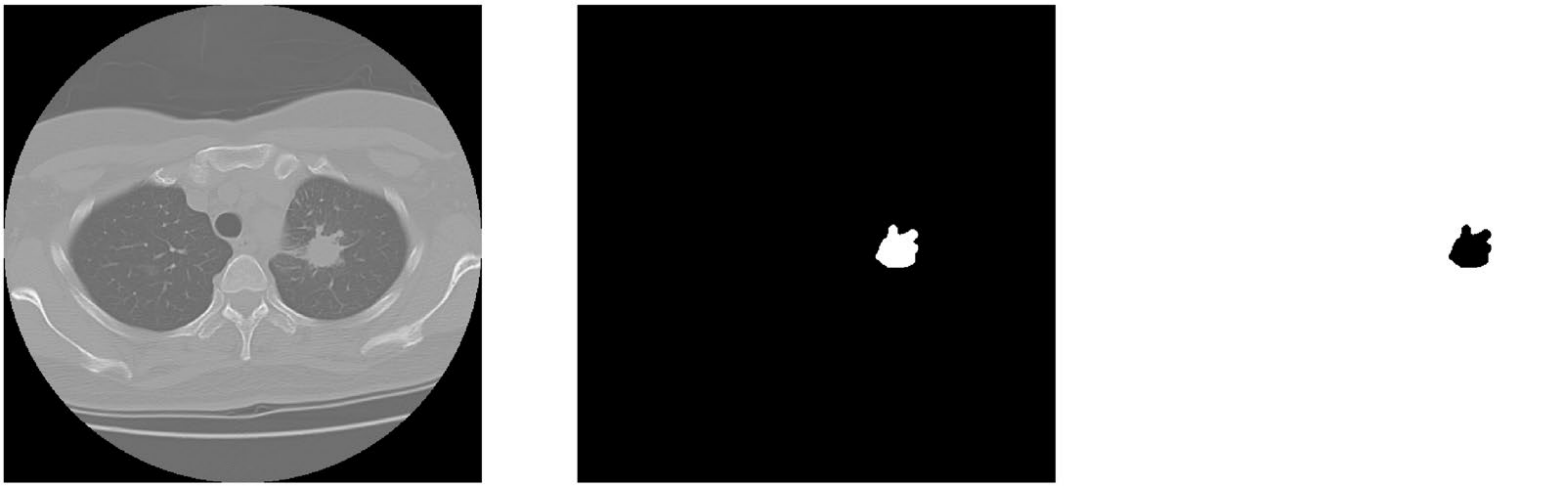
Inverting the pulmonary label. From left to right, CT image, original label, complementary label.

Three different kinds of model labeling inputs, original labeling, complementary labeling, and hybrid labeling are considered in this study. Imbalanced data labeling is a big problem in image segmentation. We thought that using complementary labeling may get better result. Because of the exchanging between hard examples and easy examples. In clinical medical images, the area of lesion always gets a lower percentage in the whole CT image. To invert the labeling is to let the labeling area become a higher percentage in the whole CT image as shown in Fig. 6. In this paper^19^, a method like complementary labeling is proposed. The different between the paper and ours is the using of labeling. In their study, the proposal is loss function which correct the loss calculation. The proposal of our study is the data pre-processing, which means the calculation of loss is different. And another method is hybrid labeling input. The input dimensions become two; one is the original labeling and the other is the complementary labeling. So the output would get two different mask with positive and negative labeling. The calculation of dice coefficient is respectively to each one. As the input labeling become two dimensions, the weights of per pixel decreases. Ideally, by using the same loss function, it can let the calculating in backpropagation become more objective.

## Results

### Pre-processing

The dice coefficient loss is selected as the loss function. Dice coefficient as (1) is often used in medical image segmentation^18,20^.

1 $$\begin{aligned} \text {Dice Coefficient = }\frac{2|{X}\cap {Y}|}{|X|+|Y|} \end{aligned}$$

It is usually used to calculate the similarity of two samples. Data augmentation is not employed in this experiment because the amount of data is sufficient. 2D images are considered in this study. One reason is that it can efficiently decrease the computing cost. The other one is GPU limited. In most of GPU, it is hard to train 3D models due to the space problem. We are aware that there are many other 2D or 3D models. In this work, we intent to report our study on some possible ideas to resolve problem for segmentation, especially unbalance data and small. Besides, about the model training, the training progress converged in 200 epochs for the setting of 3000 epochs in this study. The results obtained from the models are showed in the confusion matrix where the ground truth are compared with respect to the model predicted values and hence the calculation of accuracy, sensitivity, and specificity are shown as the following.

2 $$\begin{aligned} \text {Accuracy}= & {} \frac{TP+TN}{TP+FP+TN+FN},\quad {\text {Sensitivity = }\frac{TP}{TP+FN}},\quad {\text {Specificity = }\frac{TN}{FP+TN}} \end{aligned}$$

3 $$\begin{aligned} \text {TP}= & {} \text {True Positive}, \quad \text {TN = True Negative}, \quad \text {FP = False Positive}, \quad \text {FN = False Negative} \end{aligned}$$

**Figure 7:**
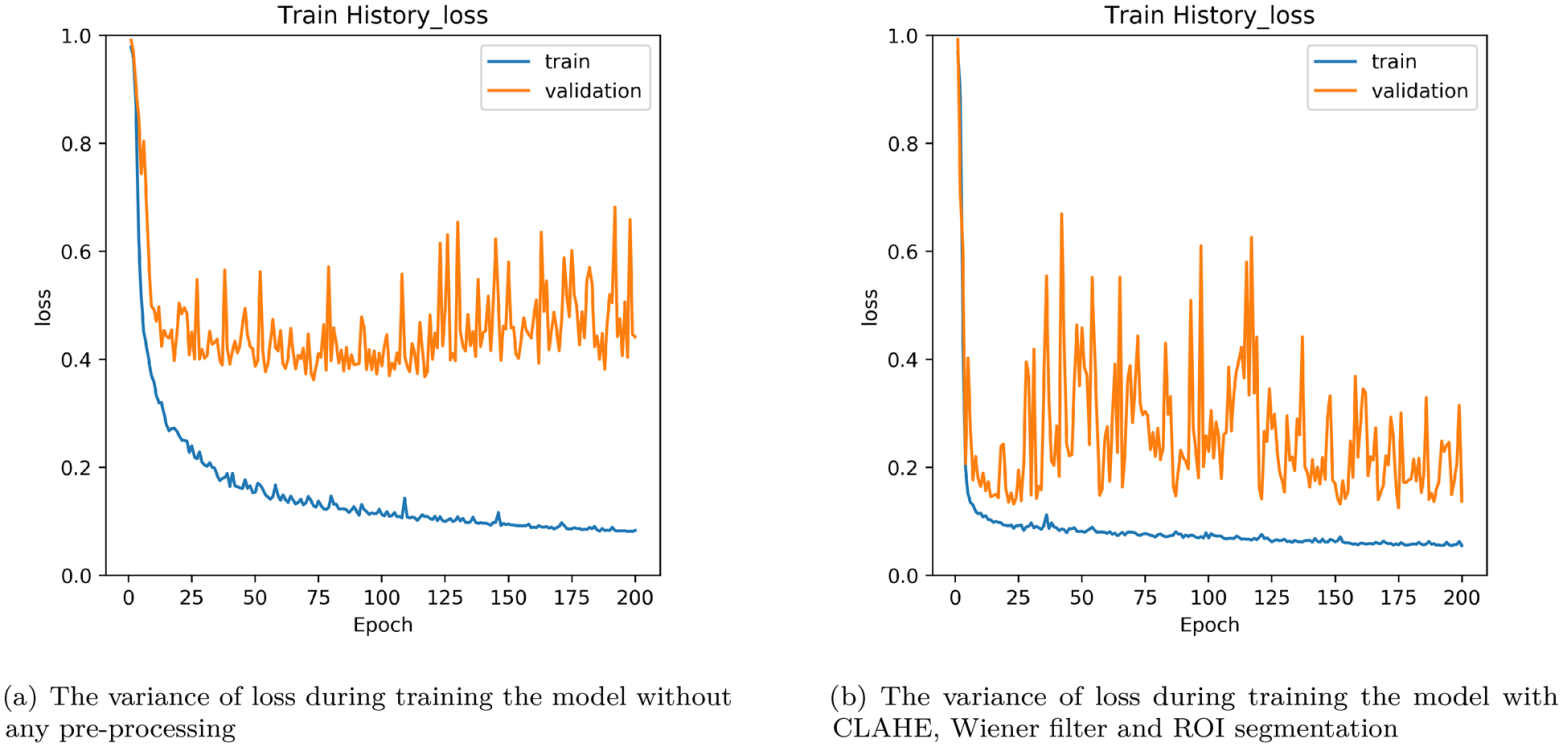
Loss variance in training model.

**Figure 8:**
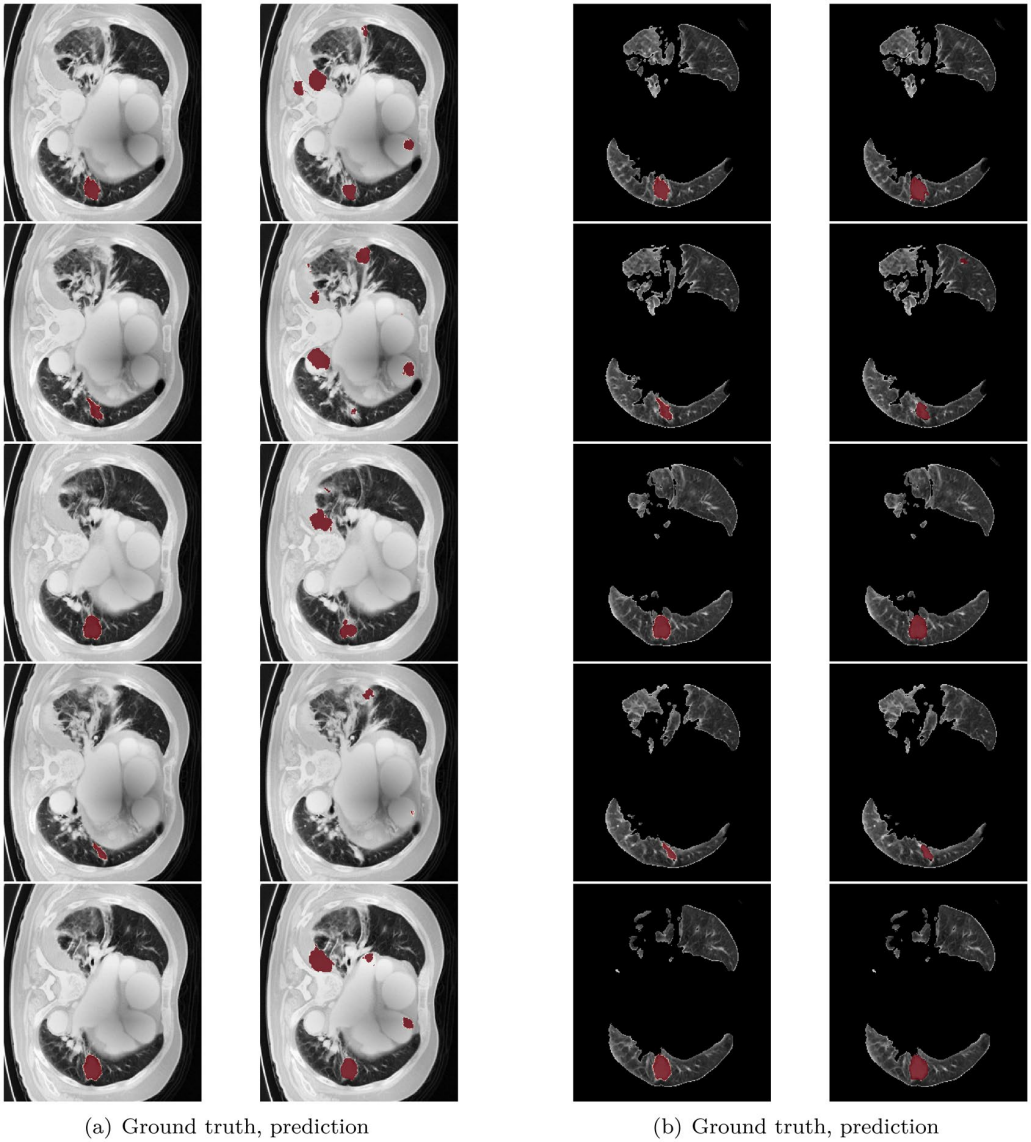
Left: With CLAHE and Wiener, Right: With CLAHE, Wiener and ROI segmentation.

As shown in Fig. 7, the testing loss under the condition without pre-processing stop to decrease around 0.4 in Fig. 7a and with ROI segmentation, CLAHE and wiener can reach as low as 0.1 in Fig. 7b. It shows that using these pre-processing methods is effective to decrease the testing loss. In order to show the effectiveness of the using of ROI segmentation, some examples of the obtained detections are shown in Fig. 8. Figure 8a are those only considering the pre-processing with CLAHE and Wiener. The left side is the ground truth and the right side is the prediction of the model. It can be observed wrong prediction figures show that the model always predicts on the non-pulmonary area. In Fig. 8b, the results by adding the approach of ROI segmentation. It is clearly evident that it can resolve the problem of the model labeled in the wrong areas. Because of that, the model trained can get lower loss. Also, it can be found that it keeps decreasing as the training epoch goes on. The training process becomes more smooth.

### Complementary labeling

As mentioned, complementary labeling is considered to train the U-net. With two dimensions of labeling use the same loss function. The model will tend to decrease the loss in two labeling dimensions. In other words, the weight of per pixel decreases. In the output, the results of two dimensional predicted output are obtained by performing interaction with them. Table 2 shows the comparison among different methods in our testing data, without pre-processing and with pre-processing (with CLAHE, wiener filter, and ROI segmentation). From the table, it can be found that the Sensitivity is improved from 0.90 to 0.96 and the dice coefficient is improved from 0.573 to 0.790 due to the pre-processing. Also, the results are considered with different labeling ways in mono or in hybrid. Mono means only one label input in positive or negative (complementary labeling) is used and hybrid means two label inputs in positive or negative output are used. It shows that whether using positive or negative ground truth as model input, the result cannot be better than using mono input significantly. It seems that using complementary labeling is not effective under the big data quantity condition.

Different data quantities, 472 cases and 50 cases are considered in this study. As shown in Table 3, it seems that using complementary labeling with pre-processing gets a better result than using mono input. But without pre-processing, complementary labeling is not efficient. It is worthwhile to use the complementary labeling method in small data quantity. That is why in this study complementary labeling is considered. The sensitivity of small data quantity, the hybrid negative is higher than using mono input positive with the pre-processing conditions. The hybrid negative is 0.9716 and the mono input positive is 0.9560. Thus, if data quantity is not big enough, trying to use complementary labeling is a good method to get better result.

### ROI segmentation model

It is noted that the ROI segmentation model used in this study is different from other approaches like these in the literature^21,22^. In those approaches, 3D ROI segmentation methods are employed to get the position of pulmonary. However, in this study, only a 2D segmentation method is considered. Although the result of our approach is not very good, there is future work need to do. The reason for the results being not good enough is the segmentation is not perfect in small lung slices. The nodules seldom appear in the top or bottom of the pulmonary. And the segmentation effects in those positions are not perfect. So the U-net, prediction model cannot get good results in these slices. Transfer learning is necessary to do in future work from 3D ROI segmentation methods. Nevertheless, by using this method in TMUH dataset can efficiently increase the dice coefficient score. The model can also widely be used in other CT lung images. Compared with the traditional method, ROI segmentation model can have better results even on 2D segmentation.

**Table 2:** The comparison of different method in lung nodule segmentation.

Evaluation	Without pre-processing	With pre-processing (mono input positive)	With pre-processing (mono input negative)
Dice coefficient	0.573	0.790	0.701
Confusion matrix	Accuracy:0.9986	Accuracy:0.9997	Accuracy:0.9997
	Sensitivity:0.9033	Sensitivity:0.9614	Sensitivity:0.9724
	Specificity:0.9987	Specificity:0.9998	Specificity:0.9997

Evaluation	With pre-processing (hybrid positive output)	With pre-processing (hybrid negative output)	
Dice coefficient	0.744	0.639	
Confusion matrix	Accuracy:0.9996	Accuracy:0.9994	
	Sensitivity:0.9502	Sensitivity:0.9650	
	Specificity:0.9996	Specificity:0.9994

**Table 3:** The comparison in small data quantity (50 cases) of different method in lung nodule segmentation.

Evaluation	With pre-processing (mono input positive)	With pre-processing (hybrid positive)	With pre-processing (hybrid negative)
Dice coefficient	0.622	0.608	0.628
Confusion matrix	Accuracy:0.9984	Accuracy:0.9967	Accuracy:0.9959
	Sensitivity:0.9560	Sensitivity:0.9561	Sensitivity:0.9716
	Specificity:0.9984	Specificity:0.9967	Specificity:0.9959

Evaluation	Without pre-processing (mono input positive)	Without pre-processing (hybrid positive)	Without pre-processing (hybrid negative)
Dice coefficient	0.304	0.228	0.225
Confusion matrix	Accuracy:0.9961	Accuracy:0.9904	Accuracy:0.9898
	Sensitivity:0.4735	Sensitivity:0.1431	Sensitivity:0.1557
	Specificity:0.9964	Specificity:0.9909	Specificity:0.9904

## Discussion

We have shown that using complementary labeling is efficient in small data quantity in this study. However, using the pre-processing method such as CLAHE and ROI segmentation is also efficient as shown in our experiments. The results obtained by using some pre-processing method and complementary labeling show good results as shown in Table 3. In addition, although the testing dataset is only 4 cases. As shown in Table 4, the variance of mean and std in different dataset is not disparate obviously. Because the testing data is split from the same source to training data and validation data. To conclude, the model trained is U-net and the loss function used is dice coefficient loss. As the result shows in Tables 2 and 3, complementary labeling does not show good effects with a big quantity of data but show better results in a small quantity of data. The main reason is the background (no nodule) is more easily to be found than the foreground (nodule) in a small data set, and the foreground is more easily to be found than the background in a big data set. There are only 50 cases of patient CT images at first in this study. Due to this reason, we choose complementary labeling somewhat like a data augmentation method to train the model. Using ROI segmentation is efficient, but in some image slices (no lung) are not efficient. The model tends to predict something in no lung image slices. The main idea of this study is to use a 2D ROI segmentation model to get the position of pulmonary. In future work, transfer learning is necessary to do to make this model more completed. Although the result of complementary labeling is not efficient in a big quantity of data, the consumption of computing power and time is good. Using an NVIDIA RTX 2080 Ti GPU with 11 GB RAM, training took almost 3 days for 3000 epochs. Future research should do the data augmentation if the GPU memory is enough. In the paper^23^, using a large batch can get better results than use a small batch. Using 3D images will consume more memory in GPU. How to find a trade-off between the input image size and the batch size is an important issue to be considered.

**Table 4:** The mean and std of loss in original data quantity training.

Data	Testing	Validation	Each testing data			
			1	2	3	4
Mean	0.7901	0.7885	0.9204	0.5174	0.5478	0.9071
Std	0.3276	0.2984	0.0125	0.3668	0.4487	0.1916
